# Dual-functional cellulase-mediated gold nanoclusters for ascorbic acid detection and fluorescence bacterial imaging

**DOI:** 10.3389/fbioe.2023.1258036

**Published:** 2023-08-28

**Authors:** Baojuan Wang, Jinxin Fang, Huiliang Tang, Shan Lu, Yan Chen, Xiaoqi Yang, Yuezhen He

**Affiliations:** ^1^ Anhui Provincial Key Laboratory of Molecular Enzymology and Mechanism of Major Diseases, College of Life Sciences, Anhui Normal University, Wuhu, Anhui, China; ^2^ Key Laboratory of Biomedicine in Gene Diseases, Health of Anhui Higher Education Institutes, College of Life Sciences, Anhui Normal University, Wuhu, Anhui, China; ^3^ Anhui Key Laboratory of Chemo-Biosensing, Ministry of Education, Anhui Normal University, Wuhu, China; ^4^ Key Laboratory of Functional Molecular Solids, Ministry of Education, Anhui Normal University, Wuhu, China; ^5^ Laboratory of Biosensing and Bioimaging (LOBAB), College of Chemistry and Materials Science, Anhui Normal University, Wuhu, China

**Keywords:** Au nanoclusters, biomineralization, fluorescence, biosensor, bacterial labeling

## Abstract

Protein-protected metal nanomaterials are becoming the most promising fluorescent nanomaterials for biosensing, bioimaging, and therapeutic applications due to their obvious fluorescent molecular properties, favorable biocompatibility and excellent physicochemical properties. Herein, we pioneeringly prepared a cellulase protected fluorescent gold nanoclusters (Cel-Au NCs) exhibiting red fluorescence under the excitation wavelength of 560 nm via a facile and green one-step method. Based on the fluorescence turn-off mechanism, the Cel-Au NCs were used as a biosensor for specificity determination of ascorbic acid (AA) at the emission of 680 nm, which exhibited satisfactory linearity over the range of 10–400 µM and the detection limit of 2.5 µM. Further, the actual sample application of the Au NCs was successfully established by evaluating AA in serum with good recoveries of 98.76%–104.83%. Additionally, the bacteria, including gram-positive bacteria (*Bacillus subtilis and Staphylococcus aureus*) and gram-negative bacteria (*Escherichia coli*), were obviously stained by Cel-Au NCs with strong red emission. Thereby, as dual-functional nanoclusters, the prepared Cel-Au NCs have been proven to be an excellent fluorescent bioprobe for the detection of AA and bacterial labeling in medical diagnosis and human health maintenance.

## Introduction

Ascorbic acid (AA, vitamin C), as one of the most vital micronutrients and antioxidants in the human body, plays an imperative role in numerous biochemical reactions involving oxidative stress reduction, disease prevention, immune response and other physiological activities (Abulizi et al., 2014; Liu et al., 2017). Furthermore, AA is also a medicine for the treatment of many diseases, including scurvy, immunodeficiency, allergic reactions and liver disease, which contributes to the absorption of iron and calcium, healthy cell development, and normal tissue growth (Zhuang and Chen, 2020). Thus, AA detection is very important in medical diagnosis and human health maintenance. At present, various analytical methods have been developed and utilized in the quantitative determination of AA, such as electrochemistry (Ma et al., 2021), high liquid chromatography (Burini, 2007), liquid chromatography-mass spectrometry/mass spectrometry (Diep et al., 2020). Although these technologies have been successfully implemented in AA detection, most of them still have disadvantages such as complicated instrument requirements, long detection time, and low sensitivity. Nowadays, the fluorescence method has gradually become an ideal alternative method for detecting AA because of its simplicity, high sensitivity and excellent reproducibility (Gan et al., 2020). Therefore, it is urgent to develop an innovative material with exceptional fluorescent properties in biosensing.

Metal nanoclusters (NCs) consisting of several to dozens of atoms are typically ∼3 nm which is equivalent to the Fermi wavelength of the electrons (Jin et al., 2016), resulting in a series of tunable metal core composition with discrete electronic states, obvious fluorescence molecular-like characteristics and excellent physicochemical properties (Zhang and Wang, 2014). Due to their inherent properties, metal NCs including Au, Ag, Cu, Pd and Pt NCs are being widely explored in biological imaging, biological sensing and advanced therapeutics fields (Guo et al., 2021; Tan et al., 2021). Notably, Au NCs become the most promising fluorescent nanomaterial owing to their excellent characteristics, such as strong photoluminescence, extraordinary photostability, explicit composition and combination properties (Guo et al., 2021). In light of this, various methods including microwave-assisted synthesis (Yue et al., 2012), sonochemistry (Xu and Suslick, 2010), photoreduction (Zhou et al., 2017), ligand-induced etching (Duan and Nie, 2007), and template-assisted synthesis (Qiao et al., 2021; Chen et al., 2022) have been developed to form the Au NCs.

Up to now, many templates, including DNA, proteins, viruses, microorganisms and plants, have been used for the preparation of Au NCs (Huang et al., 2015; Chen et al., 2018; Wang et al., 2019). Among them, due to their specific amino acid sequence composition, unique spatial conformation and chemical functional groups, proteins as an effective biological template show tremendous potential for the synthesis of Au NCs with tunable size, fluorescent properties and favourable biocompatibility (Yu et al., 2014; Guo et al., 2021). For example, Bhamore et al. prepared amylase Au NCs with red fluorescent emission and an average size of 1.75 nm for the detection of deltamethrin and glutathione (Bhamore et al., 2019). In another case, human serum albumin (HSA) directed red-emitting gold nanoclusters (HSA-AuNCs) were used as a bioprobe for *Staphylococcus aureus* (Chan and Chen, 2012). Moreover, in our recent study, using flavourzyme as a template, first prepared Fla-Au NCs with blue fluorescence were successfully utilized for the determination of carbaryl (Chen et al., 2022). Papain-encapsulated platinum nanoclusters with green fluorescence can be used not only for sensing lysozyme in biofluids but also for gram-positive bacterial identification (Chang et al., 2021). Therefore, it is urgent to develop innovative protein-coated metal nanoclusters and explore their applications in bioprobes, bioimaging and therapy.

Cellulase (Cel), as a pivotal industrial enzyme, catalyzes the decomposition of renewable lignocellulosic biomass into oligosaccharides or monosaccharides, which have been explored in numerous industries, such as textile, pulp and paper, detergent, food, and biofuel production (Ejaz et al., 2021; Areeshi, 2022). However, there are very limited reports on the synthesis and application of cellulase mediated nanostructure. Up to now, only Cel-protected copper nanoclusters (Cu NCs) with exceptional photostability, luminescence quantum yield, and colloidal stability has been investigated (Singh et al., 2016). Additionally, attributed to the oxidation resistance, conductivity, non-toxicity and stability of Au, the performance of Au NCs in biosensing and biomedicine is highly anticipated.

**SCHEME 1:**
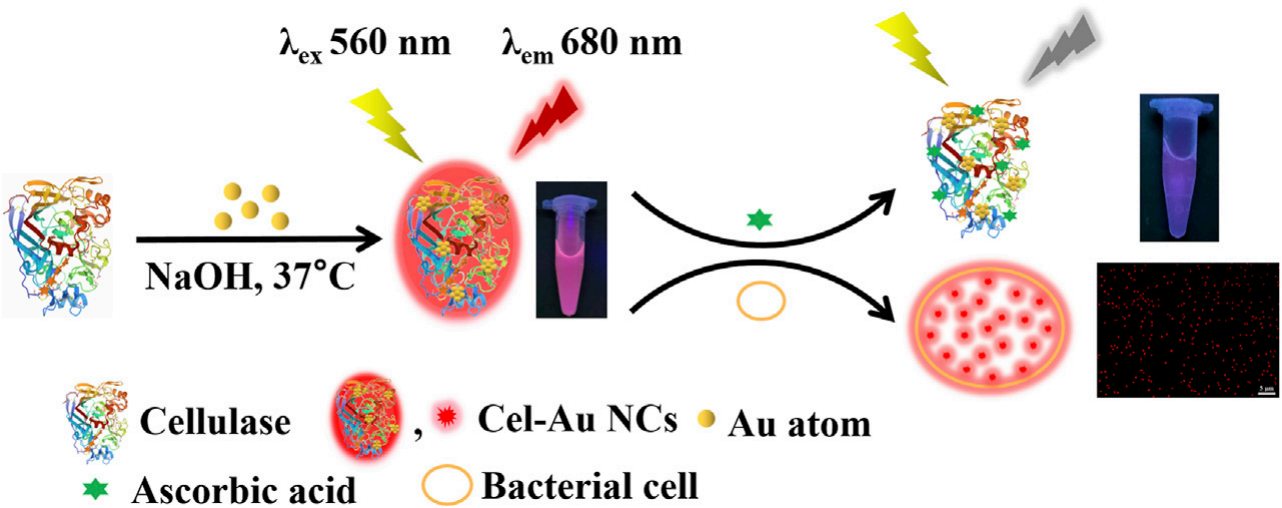
Schematic illustration of Cel-Au NCs for sensing ascorbic acid and bacterial labeling.

Hereby, we innovatively fabricated one type of red-emitting Au NCs using cellulase as the template via a one-step biomineralization method. A series of characterization techniques were used to explore the optical properties, morphology, composition, and valence state of Cel-Au NCs, including UV-vis absorption spectrometry, fluorescence spectroscopy, transmission electron microscopy (TEM), Fourier transform infrared spectroscopy (FT-IR) and X-ray crystallography (XPS). As shown in Scheme 1, this turn-off and label-free biosensor provided an alternative choice for AA detection in the biofluid. Meanwhile, owing to ultra-small size, brightly red fluorescence and good biocompatibility, dual-functional Cel-Au NCs could also be served as a bio-imaging probe for bacterial imaging.

## Materials and methods

### Materials

HAuCl_4_^.^4H_2_O was purchased from Sinopharm Chemical Reagent Co., Ltd. (Shanghai, China). Cellulase, pepsin, trypsin and AA were obtained from Yuanye Biotechnology Co., Ltd. (Shanghai, China). Histidine, threonine, lysine, glycine, glutathione (GSH), maltose, sucrose, glucose and metal ions were acquired from Sangon Biotechnology Co., Ltd. (Shanghai, China). All reagents were of analytical purity and used directly. Milli-Q purified water prepared by the PR03200 ultra-pure water meter (Zhongshan Keningte Cleaning Supplies Co., Ltd.) was utilized in all experiments.

### Instruments

All glass containers in the laboratory were thoroughly washed with aqua regia, rinsed with ultrapure water and dried before use. UV-1800 spectrophotometer (Shimadzu, Japan), PF-5301PC fluorescence spectrophotometer (Shimadzu, Japan) and Spark-Multimode microplate reader (Tecan, Switzerland) were applied to measure the UV-vis absorption spectra, the fluorescence spectra, and the bacterial density, respectively. Transmission electron microscopy (TEM) images were collected on a JEOL 2010 LaB6 TEM (TECNAI G2, the Netherlands) at an acceleration voltage of 200 kV. Fourier transform infrared (FT-IR) spectra and X-ray photoelectron spectra (XPS) were separately detected by BW17-FTIR-650 spectrometer (Beijing, China) and X-ray photoelectron spectroscopy (Shimadzu, Japan). The fluorescence lifetime and quantum yield (QY) of the samples were recorded on an FLS920 fluorescence spectrometer (Edinburgh, UK). Zeta potential values were performed using the Malvern Zetasizer sizer NanoZS ZEM-3600 instrument (Malvern, UK). Furthermore, bacteria imaging was collected using the fluorescence microscope (Zeiss, Germany).

### Synthesis of Cel-Au NCs

Typically, 0.16 mL of the HAuCl_4_ solution (25 mM) and 9.84 mL of the cellulase solution (1 mM) were mixed thoroughly with a vortexer for 5 min. After adjusting pH to 12 with the addition of 1 M NaOH solution, the above mixture was reacted at 37°C for 12 h in the dark. Then the supernatant of the above mixture was collected by centrifugation at 8,000 rpm for 10 min, dialyzed to remove unreacted metal ions by a dialysis membrane (1, 000 MWCO) for 24 h, and placed at 4°C for future use.

### The detection of AA

For AA detection, the Cel-Au NCs (40 mg/mL, 50 μL), different concentrations of AA solutions (100 μL) and deionized water (850 μL) were mixed and incubated at 25°C for 5 min in a water bath. The fluorescence signal of the above mixture was then measured using an F-4500 fluorescence spectrophotometer by exciting at 560 nm. To evaluate the selectivity and specificity of Cel-Au NCs for AA, the fluorescence variations of Cel-Au NCs were investigated toward 16 kinds of compounds (histidine, threonine, lysine, glycine, GSH, maltose, sucrose, glucose, AA, KCl, NaCl, LiCl, ZnCl_2_, CaCl_2_, MgCl_2_, MnCl_2_). The as-prepared Cel-Au NCs were mixed with different compound solutions and measured under the same experimental condition as above. All experiments were performed three times in a parallel format.

### Analysis of AA in real samples

To evaluate the applicability of the method, human serum samples provided from the Hospital of Traditional Chinese Medicine (Wuhu, China) were directly diluted 40 times with Milli-Q purified water before the experiment. Then, 50 μL of 40 mg/mL as-prepared Cel-Au NCs, 850 μL of diluted serum sample and 100 μL of different concentrations of AA solution were mixed and analyzed in accordance with the procedure mentioned above.

### Bacterial culture and viability assay

*Bacillus subtilis* (*B. subtilis*, gram-positive bacteria), *Staphylococcus aureus* (*S. aureus*, gram-positive bacteria) and *Escherichia coli* (*E. coli*, gram-negative bacteria) were separately cultured on Luria-Bertani (LB) agar plates at 37°C overnight. Subsequently, a single colony of the bacteria was separately picked and incubated in LB liquid culture medium with continuous shaking at 180 rpm at 37°C for another 16–24 h.

To estimate the biocompatibility of Cel-Au NCs, bacterial viabilities were measured by determining bacterial cell density at OD_600_ on Spark-Multimode microplate reader. When OD_600_ reached 0.6, the bacteria (*B. subtilis*, *S. aureus*, and *E. coli*) were seeded into a 96-well microplate at 1% inoculum. Then various concentrations of Cel-Au NCs (0, 10, 25, 50, 100 and 200 μg/mL) were separately added to the bacteria and cultured at 37°C and 180 rpm. The growth of organisms was observed by measuring OD_600_ until 24 h and all of the experiments were executed three times in parallel. The percentage of bacterial density without adding Au NCs was taken as 100%.

### Fluorescent imaging of bacteria

After centrifuging at 8,000 rpm for 5 min, the above cultured bacterial cells were collected, washed with PBS, and incubated in the mixture of the prepared Cel-Au NCs (0.1 mL) and PBS (0.4 mL) in a shaker at 37°C for 15 min. The bacterial cultures were examined on a Zeiss upright fluorescence microscope under 605 nm.

## Results and discussion

### Synthesis and characterization of Cel-Au NCs

The red-emitting Cel-Au NCs were firstly prepared via a facile and green one-step biomineralization method based on the reduction of cellulase provided by sulfur-containing cysteines and methionines, which made the Au-S band formed between cellulase and Au atom (Balu et al., 2019; Wang et al., 2019). To obtain the optimal conditions of the synthesized Cel-Au NCs, the molar ratio (cellulase/HAuCl_4_) and reaction pH were conducted in Supplementary Figure S1. The molar ratio of 2.5:1 and the reaction pH of 12 served as optimal conditions were selected for further study.

**FIGURE 1:**
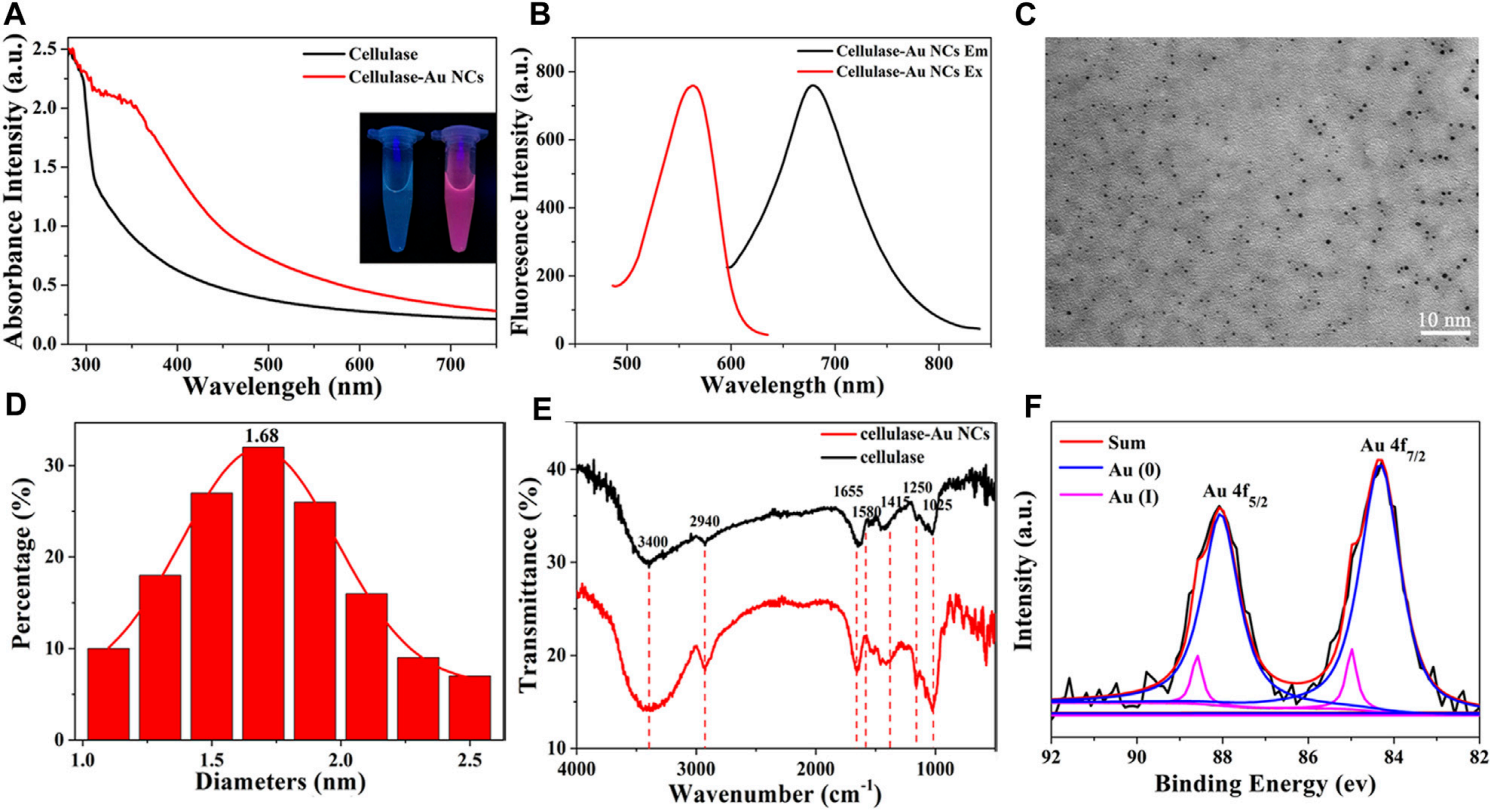
**(A)** UV-vis absorption spectra of Cel-Au NCs (red line) and cellulase (black line), Inset: photographs of cellulase (left) and Cel-Au NCs (right) with UV light (365 nm). **(B)** Fluorescence excitation spectra of Cel-Au NCs at emission wavelength 680 nm (red line) and emission spectra of Cel-Au NCs upon excitation at 560 nm (black line). **(C)** Transmission electron microscopy images showed the average size of Cel-Au NCs with 10 nm bar. **(D)** Size distribution histogram of Cel-Au NCs calculated from the TEM images by counting 146 samples. **(E)** FT-IR spectra of cellulase-Au NCs (red) and cellulase (black). **(F)** XPS spectra for the Au 4f of Cel-Au NCs. The original spectrum is black, the fitted spectrum is red, the Au(0) 4f_7/2_ spectrum is blue, and the Au(I) 4f_7/2_ spectrum is pink.

Initially, UV-vis absorption spectra and fluorescence spectroscopy were employed to identify related optical properties of Cel-Au NCs. The UV-vis spectrum showed that Cel-Au NCs had a shoulder peak in the region range of 300–400 nm with a continuous rise and a distinct peak at 350 nm attributed to oxidation between cellulase and Au atoms, whereas the spectrum of cellulose showed no peak in these ranges, signifying the Cel-Au NCs were fabricated (Figure 1A). As shown in Figure 1B; Supplementary Figure S2, the red-emitting cellulase protected Au NCs displayed an emission peak maximum at 680 nm upon 560 nm excitation with a marked Stokes shift of 120 nm. Additionally, the QY of Cel-Au NCs in aqueous solution was determined to be 10.19% using Rhodamine 6 G as a reference (Supplementary Figure S3).

The morphology of the prepared Cel-Au NCs was characterized by TEM, revealing that the Cel-Au NCs had a good dispersion and the average size was 1.68 nm by counting 146 samples (Figures 1C, D), which was consistent with the diameter of metal NCs prepared in previous studies (Wei et al., 2010; Bhamore et al., 2019). Subsequently, FT-IR was used to characterize the chemical composition of Cel-Au NCs. As shown in Figure 1E, the peaks of pure cellulase and Cel-Au NCs for O-H stretching, C-H stretching, C=O stretching, C-H bending, N-H stretching and C=C bending were separately observed at 3,400 cm^-1^, 2,940 cm^-1^, 1,655 cm^-1^, 1,415 cm^-1^, 1,250 cm^-1^ and 1,025 cm^-1^, whereas a distinct peak in the spectrum of Cel-Au NCs was observed at 1,580 cm^−1^ ascribing to the formation of a bond between Au and cellulose.

XPS was used to measure the oxidation states of gold in Au NCs and it showed the peaks of Au, S, C, N and O in the XPS spectra (Supplementary Figure S4). Two peaks centered at 88.0 and 84.3 eV were separately ascribed to 4f_5/2_ and 4f_7/2_ for Au (Figure 1F). The peak of 4f_5/2_ of the prepared Cel-Au NCs was further deconvoluted into two different components, one at 88.05 eV corresponding to Au (0), and the second one at 88.60 eV attributed to Au (I). Also, the two peaks of 4f_7/2_ assigning to 84.32 and 84.99 eV showed the simultaneous presence of Au (0) and Au (I) in Cel-Au NCs. The spectra of Au 4f_7/2_ showed a binding energy of > 84.0 eV, indicating both Au (0) and Au (I) existed in Cel-Au NCs and the presence of Au-S complexes formed by the formation of charge transfer bands (Bothra et al., 2017).

### Fluorescence quantification assay of AA

**FIGURE 2:**
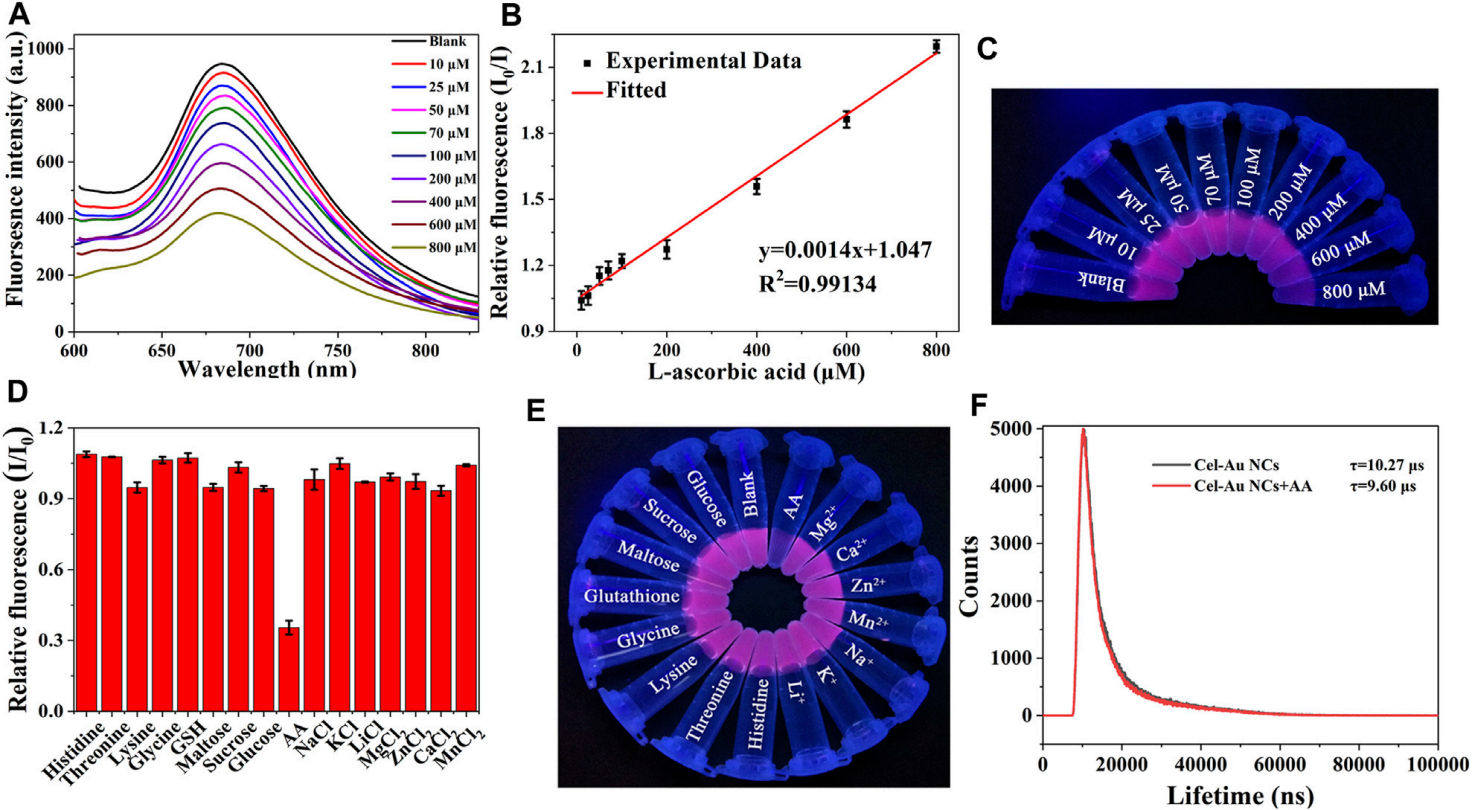
**(A)** Fluorescence spectra of Cel-Au NCs with varied concentrations of AA (top to bottom: 10–800 μM). **(B)** The linear relationship between I0/I and different concentrations from 10 to 800 μM of AA (I_0_/I, where I_0_ and I are the fluorescence intensity of Cel-Au NCs in the absence and presence of AA, respectively). **(C)** Image of Cel-Au NCs with different concentrations (10–800 μM) of AA under UV light. **(D)** Relative fluorescence intensity (I/I_0_) of Cel-Au NCs when excited at 560 nm with various analytes (I/I_0_, where I and I_0_ are the fluorescence intensity of Cel-Au NCs in the presence and absence of various analytes, respectively). **(E)** Photographic image of Cel-Au NCs solution upon the addition of various analytes under UV light illumination at 365 nm. **(F)** Time-resolved fluorescence spectra of Cel-Au NCs in the presence or absence of 600 μM AA (*λ*_ex_ = 560 nm, *λ*_em_ = 680 nm).

When the addition of AA was increased from 10 μM to 800 μM, a corresponding reduction in the fluorescent signal of Cel-Au NCs was examined (Figure 2A). Figure 2B depicted the relationship between the fluorescent intensity of the Cel-Au NCs and the different concentrations of AA, and showed a good linear correlation over a range of 10–800 µM with a LOD of 2.5 µM (*R* ^2^ = 0.99134), indicating that the detection system possessed superior sensitivity. Simultaneously, the fluorescent intensity of Cel-Au NCs was correspondingly reduced with the increasing concentration of AA by UV light (Figure 2C). Furthermore, the specificity of the Cel-Au NCs for AA was conducted by testing the response of the biosensor prepared against other compounds. Interestingly, the fluorescent intensity of Cel-Au NCs was extremely decreased just after adding AA, whereas there were barely any changes in the presence of the other compounds (Figures 2D, E; Supplementary Figure S5). Compared with the published methods in AA detection (Table 1 and Supplementary Table S1), the proposed method displayed a wider detection range and an appreciable detection limit, which is simplicity, rapidity, efficiency and economics. Thus, as an alternative biosensor, it is potential for AA detection in the biological environment using Cel-Au NCs.

**TABLE 1:** Comparison of the determination of AA using Cel-Au NCs and other reported fluorometric methods.

Materials	Linear range (μM)	Detection limit (μM)	References
Carbon dots	100-800	50	Gan et al. (2020)
Carbon dots	50–300	1.73	Shi et al. (2021)
Nanoparticles	0-750	4.9	Sun et al. (2019)
Carbon dots	20-500	5.13	Fan et al. (2022)
Carbon Quantum Dots	600-1600	18	Li et al. (2021)
Cel-Au NCs	10-800	2.5	This work

To elucidate the quenching mechanism of AA on the Cel-Au NCs, fluorescence resonance energy transfer (FRET), inner filter effect (IFE), dynamic and static quenching as well as photoinduced electron transfer had been investigated. As depicted in Supplementary Figure S6, AA displayed a strong absorption peak at 245 nm, which did not overlap with the Cel-Au NCs emission spectrum (600–800 nm), demonstrating that the mechanism of quenching mechanism caused by AA was not FRET and IFE (Fan et al., 2022). Notably, the fluorescent lifetimes were 10.27 μs and 9.60 μs for Cel-Au NCs before and after the addition of AA, separately (Figure 2F). The noticeable change in the fluorescence lifetime of Cel-Au NCs upon the addition of AA indicated that the quenching mechanism might be dynamic quenching rather than static quenching. Similarly, the fluorescence quenching of LDH-GQD caused by Fe^3+^ was determined to be dynamic quenching due to the reduction of fluorescence lifetime from 6.45 ns to 1.21 ns (Shi et al., 2021). Furthermore, the zeta potential of Cel-Au NCs increased from −15.2 mV to −13.3 mV after adding AA (Supplementary Figure S7). The negative zeta potential of the Cel-Au NCs is attributed to the presence of carboxylic groups with negative charges on the surface of cellulase, while the apparent increase in the zeta potential of Cel-Au NCs after the addition of AA confirms that the positively charged AA was attached to the surface of the negatively charged Cel-Au NCs. Additionally, the reducing power of AA caused the alteration in the oxidation state of Au (I), localized on the surface of the Au (0) core, further leading to the fluorescence quenching of Cel-Au NCs. (Li et al., 2015; Li et al., 2017). Hence, the quenching mechanism of Cel-Au NCs might be attributed to photoinduced electron transfer and dynamic quenching mechanism.

### Application of AA detection in real samples

For assessing the practicality of the method in actual samples, the detection of AA in serum samples was carried out. As depicted in Table 2, the recovery rates of AA in actual samples were in the range of 98.76%–104.83%, and the relative standard deviations (RSD) ranged from 1.05% to 5.04%. Furthermore, to demonstrate the practicability and accuracy of this biosensor, diverse concentrations of AA in serum samples were analyzed by the commercial HPLC method (Supplementary Table S2). The recoveries of AA were between 94.24% and 102.24% with RSD of 0.13%–3.07%. These results illustrated that this developed biosensor was applicable for the detection of AA in biological samples in comparison with the HPLC method.

**TABLE 2:** The concentration of AA in 40-fold diluted serum detected using the Cel-Au NCs.

Samples	Spiked (μM)	Measured (μM)	Recovery (%)	RSD (%)
Serum 1	10	9.95 ± 0.42	99.53	4.15
	25	25.07 ± 0.76	100.29	3.05
	50	49.91 ± 1.28	99.82	2.57
Serum 2	10	10.44 ± 0.37	104.39	3.70
	25	25.26 ± 0.39	101.02	1.57
	50	49.38 ± 1.55	98.76	3.10
Serum 3	10	10.48 ± 0.23	104.83	2.25
	25	24.74 ± 1.26	98.97	5.04
	50	50.23 ± 0.53	100.47	1.05

### Biocompatibility assessment of Cel-Au NCs

The biocompatibility of Cel-Au NCs was evaluated by measuring the bacterial density at OD600. The assay was conducted on three kinds of bacteria including *B. subtilis* (gram-positive bacteria), *S. aureus* (gram-positive bacteria) and *E. coli* (gram-negative bacteria). As evidenced by Supplementary Figure S8, Cel-Au NCs exhibited a negligibly inhibitory effect on bacterial cell proliferation within the range of 0–100 μg/ml and had a slight inhibitory on bacterial cell proliferation at 200 μg/ml, indicating low cytotoxicity of the Cel-Au NCs to bacteria.

### Bioimaging for types of bacteria

**FIGURE 3:**
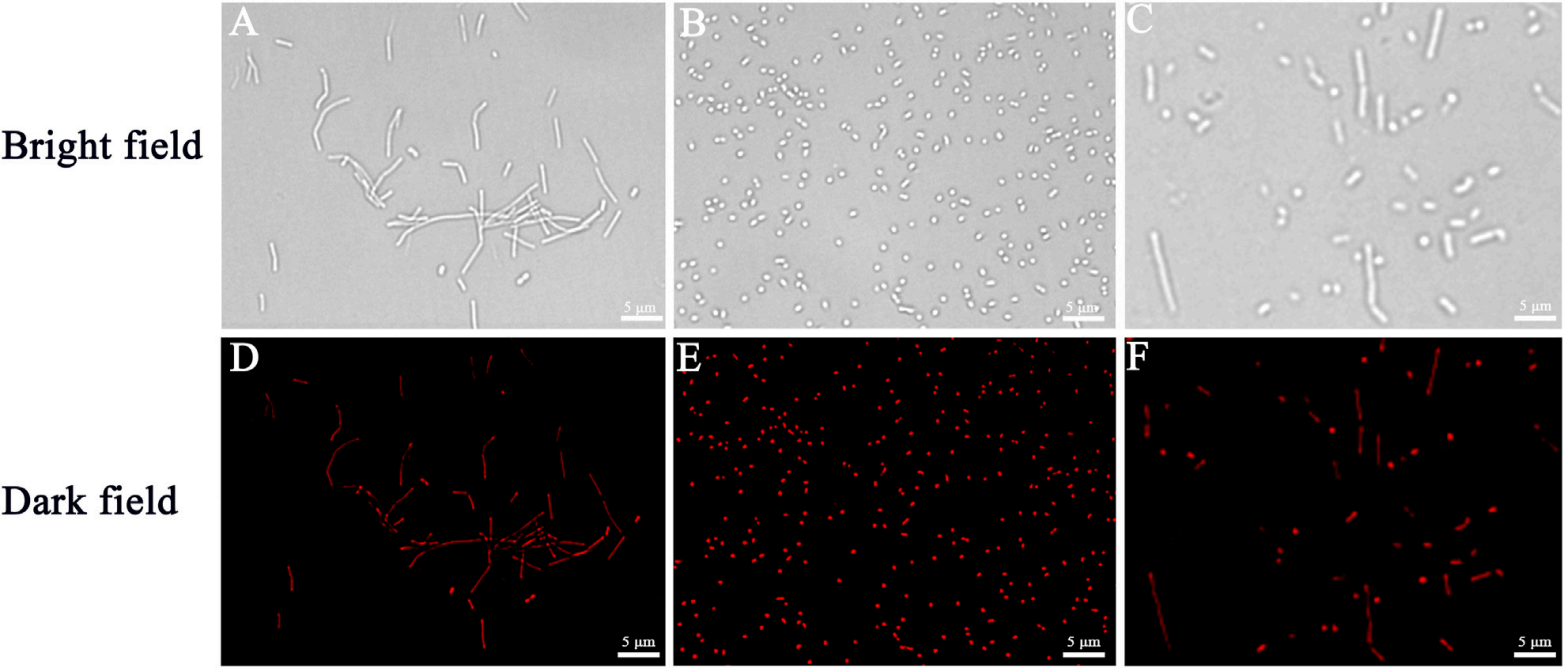
The fluorescence microscopic images of bacteria respectively correspond to bright fields and dark fields of *Bacillus subtilis* **(A,D)**, *Staphylococcus aureus* **(B,E)**, and *Escherichia coli* **(C,F)** using Cel-Au NCs as a probe.

To verify the bacterial labeling ability of Cel-Au NCs, bacterial cells incubated with Cel-Au NCs were observed under a fluorescence microscope. *B. subtilis* (gram-positive bacteria, Figures 3A, D), *S. aureus* (gram-positive bacteria, Figures 3B, E), and *E. coli* (gram-negative bacteria, Figures 3C, F) stained by Cel-Au NCs were respectively shown in the bright field and the dark field with strong red emission when excitation at 605 nm. In light of this, we hypothesized that Cel-Au NCs with ultra-small size might be absorbed by bacteria and interact with multiple proteins in the bacteria. In our previous study, *S. aureus*, *B. subtilis* as well as *Microbacterium* incubated with papain-Pt NCs could emit distinct green fluorescence (Chang et al., 2021). Besides, in the latest research, Li’s group used red-fluorescent cBSA-AuAgNCs with an average diameter of 1.80 nm to label *E. coli* (Li et al., 2022). Therefore, Cel-Au NCs with satisfactory fluorescence characteristics could be explored as a bioprobe that effectively labels the microorganism cells.

## Conclusion

In summary, with cellulase serving as the template, a one-step biomineralization strategy was successfully proposed to synthesize fluorescent Au NCs for the first time. The average size of as-synthesized Au NCs was found to be 1.68 nm and it displayed an emission peak maximum at 680 nm when excited at 560 nm. Notably, the fluorescent Cel-Au NCs as a “turn-off” biosensor could be used to assay AA with an extraordinary linear correlation over a range of 10–800 µM and a LOD of 2.5 µM. Furthermore, the practical application of the biosensor was successfully developed by evaluating AA in serum samples with appreciable recoveries of 98.76%–104.83%. In addition, Cel-Au NCs displayed a negligibly inhibitory effect on bacterial cell proliferation over 0–100 μg/ml, indicating low cytotoxicity of the pre-made Au NCs to bacteria. Furthermore, due to ultra-small size, obvious red fluorescence, and water solubility, Cel-Au NCs were also used as a bioprobe for various bacterial labeling, including *B. subtilis*, *S. aureus* and *E. coli*. This analytical and bioimaging procedure is notable as it can perform directly in a complicated environment and does not require any organic reagents as pretreatment. Therefore, this study provides new protein-directed and dual-functional Au NCs open alternative avenues for AA detection and bacterial imaging in biomedical fields.

## Data Availability

The original contributions presented in the study are included in the article/Supplementary Material, further inquiries can be directed to the corresponding authors.
