# Supplementary material for: Dual-functional cellulase-mediated gold nanoclusters for ascorbic acid detection and fluorescence bacterial imaging
Source: Front Bioeng Biotechnol. 2023 Aug 28;11:1258036. doi: 10.3389/fbioe.2023.1258036 (PMC10498280; doi:10.3389/fbioe.2023.1258036)
Supplement: Supplementary file 1 [file DataSheet1.DOCX]

**Supporting information**


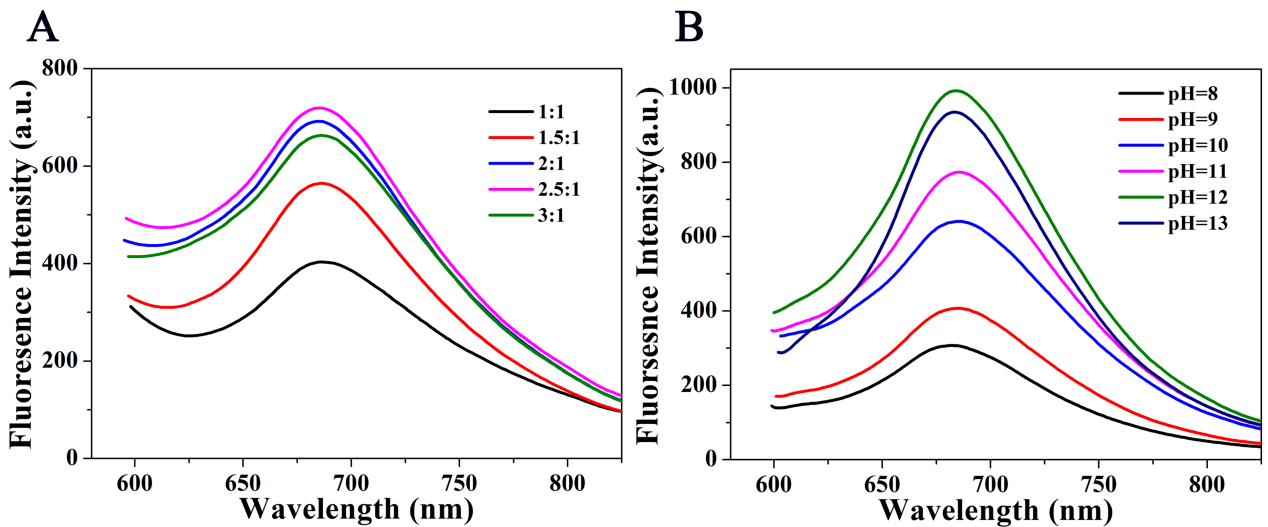


Fig. S1. Optimization of experimental conditions of Cel-Au NCs. (A) Molar ratio (Cellulase/HAuCl_4_) from 1:1 to 3:1. (B) The influence of pH from 8 to 13.


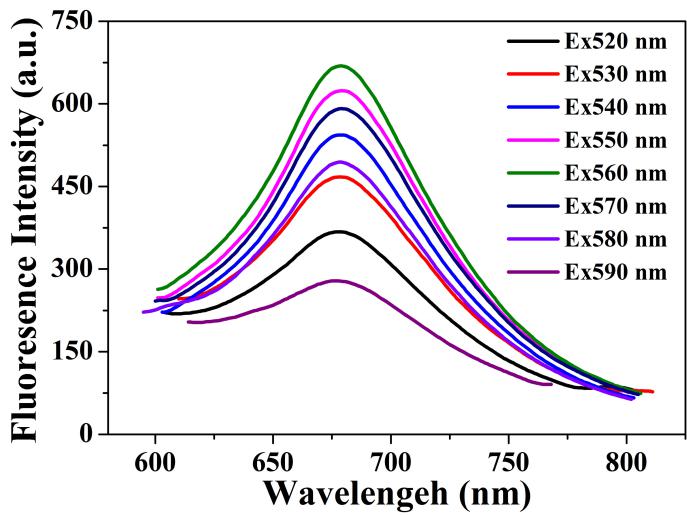


Fig. S2. The excitation-independent fluorescence spectra of Cel-Au NCs ranging from 520 nm to 590 nm.


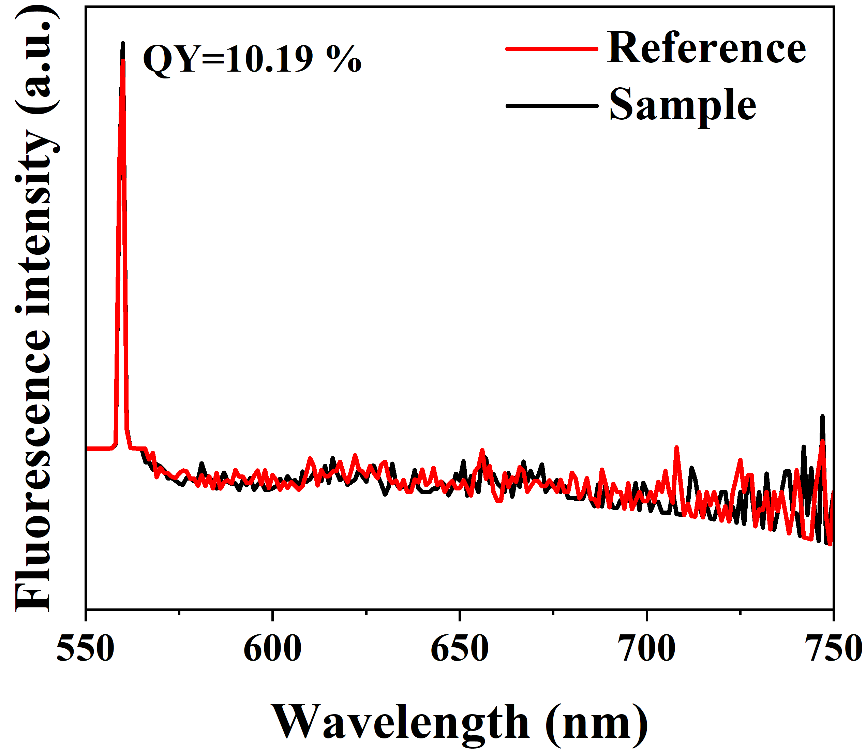


Fig. S3. Fluorescence quantum yield map of Cel-Au NCs.


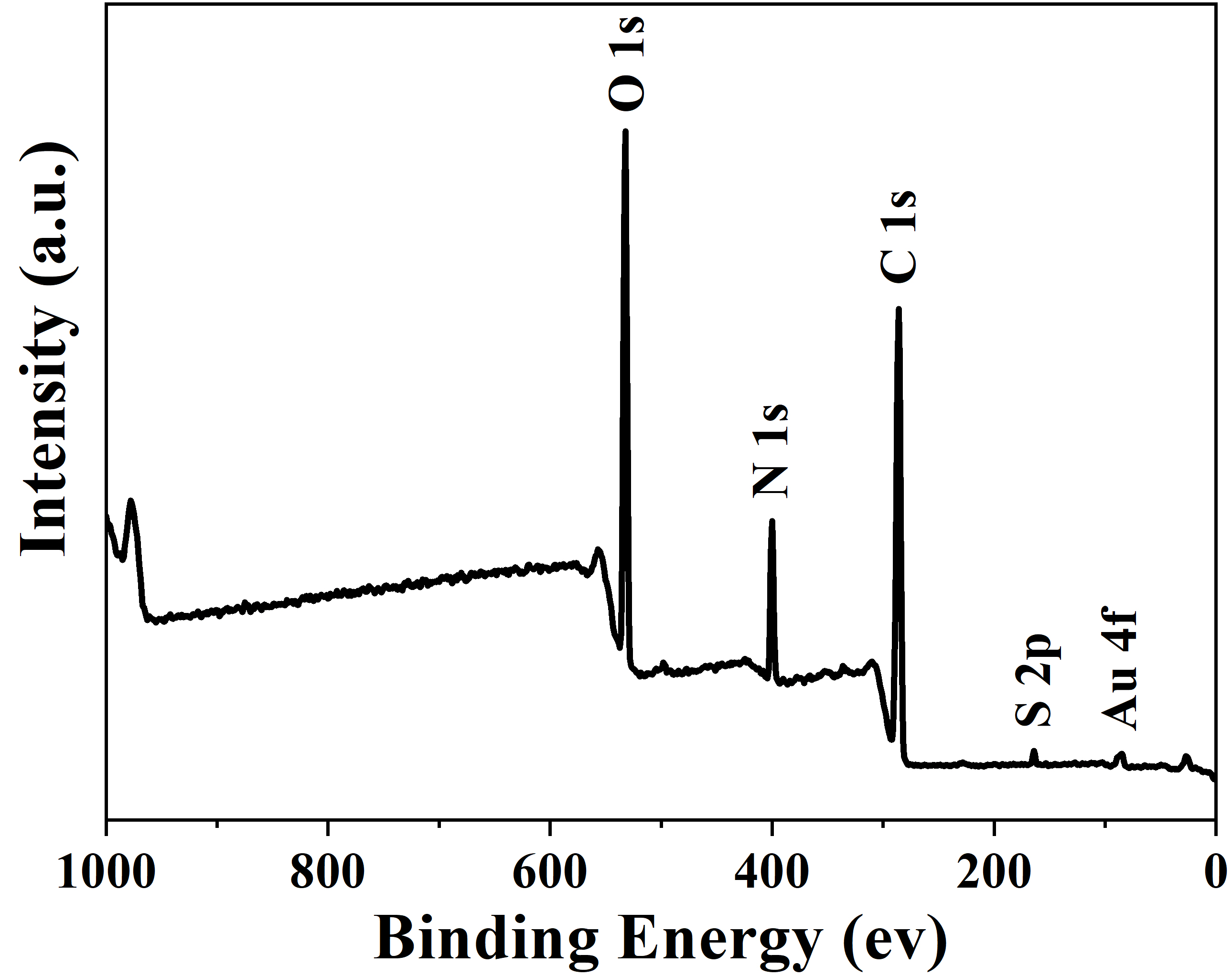


Fig. S4. XPS survey spectrum of Cel-Au NCs.


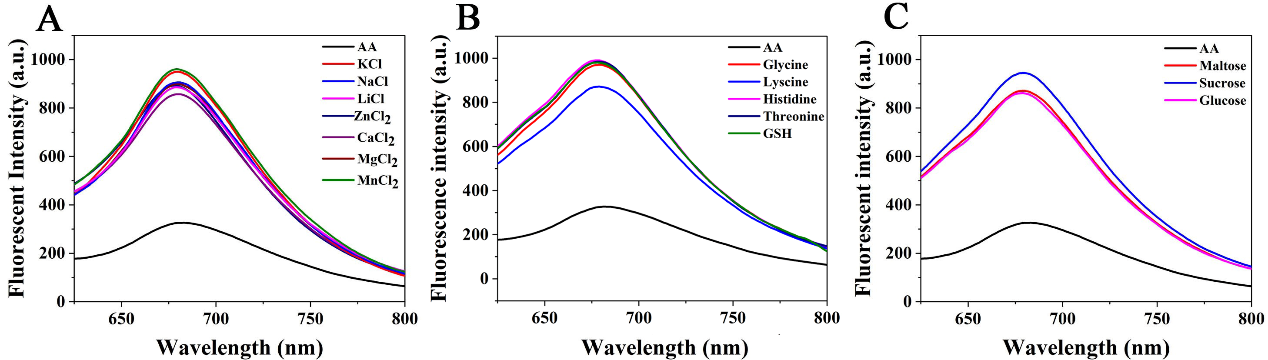


Fig. S5. Fluorescent intensity of Cel-Au NCs when excited at 560 nm with various analytes. (A) Fluorescent intensity of Cel-Au NCs with diverse metal ions (K^+^, Na^+^, Li^+^, Zn^2+^, Ca^2+^, Mg^2+^, Mn^2+^) and AA, respectively; (B) Fluorescent intensity of Cel-Au NCs with different amino acids (glycine, lysine, histidine, threonine), GSH and AA, separately; (C) Fluorescent intensity of Cel-Au NCs with various saccharides (maltose, sucrose, glucose) and AA, severally.


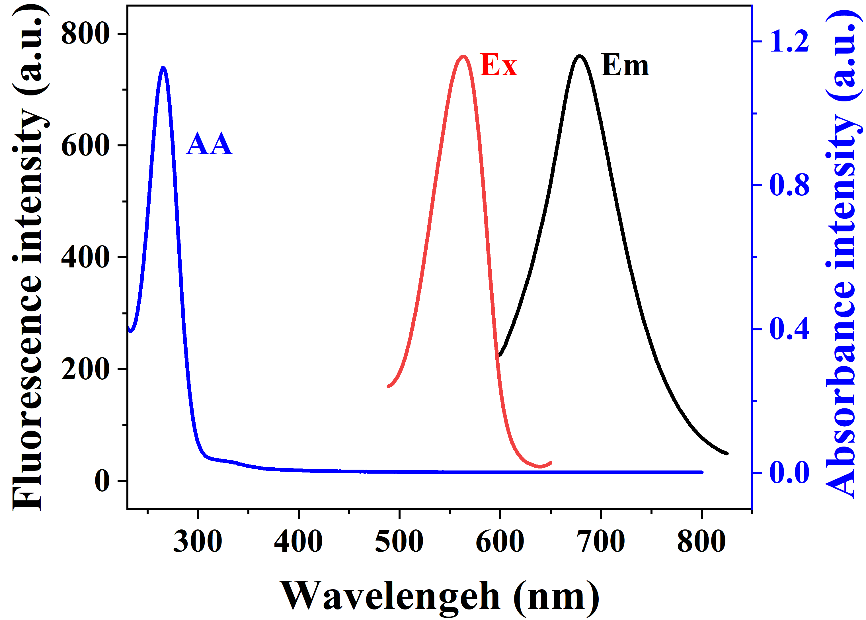


Fig. S6 Fluorescence excitation (red line, λex = 560 nm) and emission spectra (black line, λem = 680 nm) of Cel-Au NCs, and UV–vis absorption spectrum of AA (blue line).


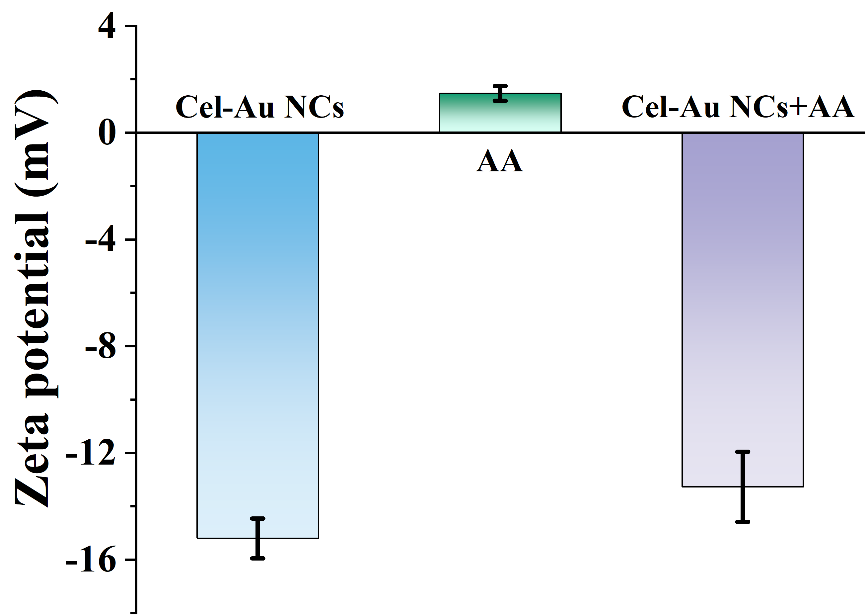


Fig. S7 Zeta potentials of Cel-Au NCs, AA, and the mixture of Cel-Au NCs and AA.


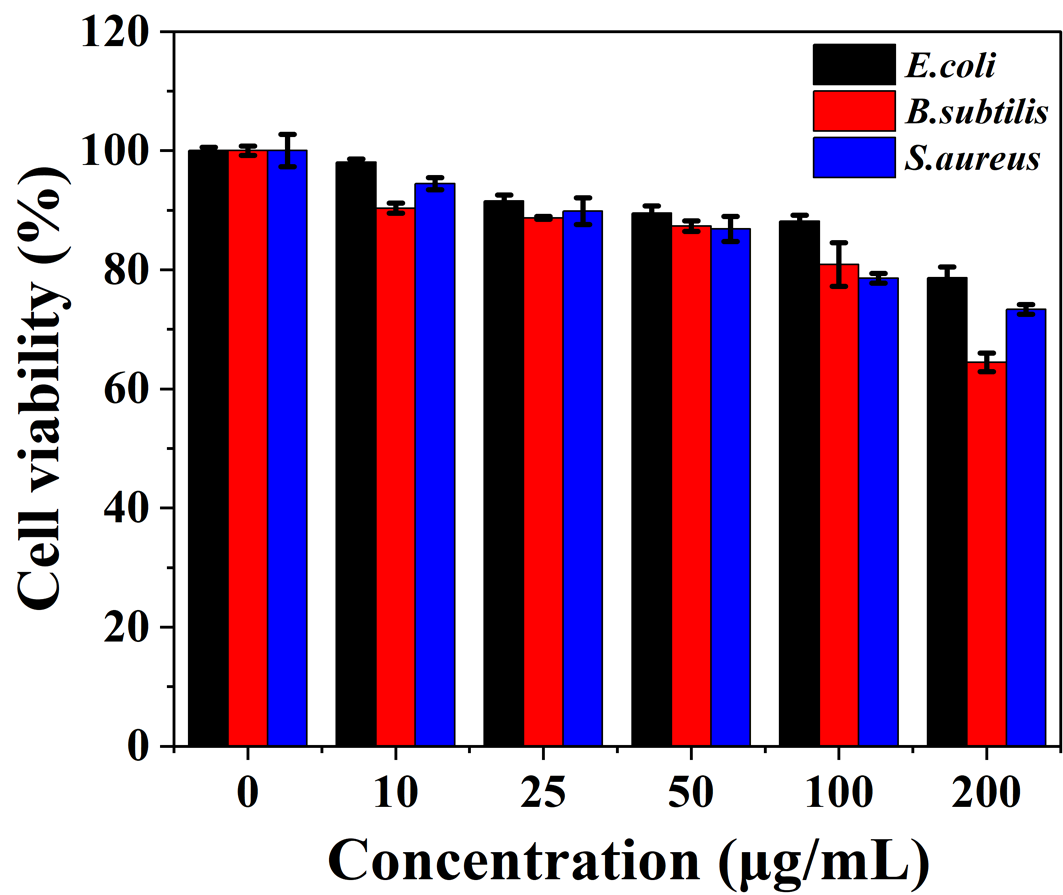


Fig. S8 Cell viability of three kinds of bacteria after treatment with Cel-Au NCs.

Table S1 Comparison of the determination of AA using Cel-Au NCs and other

reported methods.

| Methods | Materials | Linear range  (μM) | Detection  limit (μM) | References |
| --- | --- | --- | --- | --- |
| LC-MS/MS^a^ | - | 3.55-113.64 | 4.91 | (Diep et al., 2020) |
| Electrochemistry | MeGO/PANI | 8-5000 | 2 | (Naghib et al., 2020) |
| Electrochemistry | ZIF-8/Pt NPs/GCE | 10-2500 | 5.3 | (Ma et al., 2021) |
| Electrochemistry | Carbon dots | 1-1000 | <4 | (Kim et al., 2014) |
| Fluorometry | Cel-Au NCs | 10-800 | 2.5 | This work |

^a^ liquid chromatography-mass spectrometry/mass spectrometry

Table S2 The concentration of AA in serum detected using the HPLC method.

| Samples | Spiked (μM) | Measured (μM) | Recovery (%) | RSD (%) |
| --- | --- | --- | --- | --- |
| Serum 1 | 10 | 9.92 ± 0.49 | 99.17 | 2.40 |
|  | 25 | 24.25 ± 0.92 | 97.02 | 3.07 |
| Serum 2 | 10 | 9.42 ± 0.50 | 94.24 | 2.51 |
|  | 25 | 23.59 ± 0.64 | 94.36 | 2.56 |
| Serum 3 | 10 | 10.24 ± 0.25 | 102.40 | 2.50 |
|  | 25 | 24.79 ± 0.32 | 99.16 | 0.13 |

**Reference:**

Diep, T. T.; Pook, C.; Rush, E. C.; Yoo, M. J. Y. Quantification of Carotenoids, α-Tocopherol, and Ascorbic Acid in Amber, Mulligan, and Laird's Large Cultivars of New Zealand Tamarillos (*Solanum betaceum* Cav.). *Foods* **2020**, *9*, 769. DOI: [10.3390/foods9060769](https://doi.org/10.3390/foods9060769).

Kim, S. J.; Kim, Y. L.; Yu, A.; Lee, J.; Lee, S. C.; Lee, C.; Kim, M. H.; Lee, Y. Electrospun Iridium Oxide Nanofibers for Direct Selective Electrochemical Detection of Ascorbic Acid. *Sens. Actuators, B.* **2014**, *196*, 480-488. DOI: [10.1016/j.snb.2014.02.032](https://doi.org/10.1016/j.snb.2014.02.032).

Ma, Y.; Zhang, Y.; Wang, L. An Electrochemical Sensor Based on the Modification of Platinum Nanoparticles and ZIF-8 Membrane for the Detection of Ascorbic Acid. *Talanta* **2021**, *226*, 122105. DOI: [10.1016/j.talanta.2021.122105.](https://doi.org/10.1016/j.talanta.2021.122105.)

Naghib, S. M.; Behzad, F.; Rahmanian, M.; Zare, Y.; Rhee, K. Y. A Highly Sensitive Biosensor Based on Methacrylated Graphene Oxide-Grafted Polyaniline for Ascorbic Acid Determination. *Nanotechnol. Rev.* **2020**, *9*, 760-767. DOI: [10.1515/ntrev-2020-0061.](https://doi.org/10.1515/ntrev-2020-0061.)
